# Wearables and Machine Learning for Improving Runners’ Motivation from an Affective Perspective

**DOI:** 10.3390/s23031608

**Published:** 2023-02-01

**Authors:** Sandra Baldassarri, Jorge García de Quirós, José Ramón Beltrán, Pedro Álvarez

**Affiliations:** 1 Computer Science and Systems Engineering Department, Engineering Research Institute of Aragon (I3A), University of Zaragoza, 50018 Zaragoza, Spain; 2 Electronic Engineering and Communications Department, Engineering Research Institute of Aragon (I3A), University of Zaragoza, 50009 Zaragoza, Spain

**Keywords:** emotion recognition, wearable devices, machine learning, running, music recommendation

## Abstract

Wearable technology is playing an increasing role in the development of user-centric applications. In the field of sports, this technology is being used to implement solutions that improve athletes’ performance, reduce the risk of injury, or control fatigue, for example. Emotions are involved in most of these solutions, but unfortunately, they are not monitored in real-time or used as a decision element that helps to increase the quality of training sessions, nor are they used to guarantee the health of athletes. In this paper, we present a wearable and a set of machine learning models that are able to deduce runners’ emotions during their training. The solution is based on the analysis of runners’ electrodermal activity, a physiological parameter widely used in the field of emotion recognition. As part of the *DJ-Running* project, we have used these emotions to increase runners’ motivation through music. It has required integrating the wearable and the models into the *DJ-Running* mobile application, which interacts with the technological infrastructure of the project to select and play the most suitable songs at each instant of the training.

## 1. Introduction

In recent years, due to advances in the progressive miniaturisation of sensor technologies, wearables have become interesting devices in different disciplines, such as health [1] or entertainment [2,3]. However, they have become essential in the practice of many sports [4,5]. Among the wide variety of devices, smart watches, wristbands, and bracelets stand out. They allow measuring the physical activity, behaviour and performance of athletes, monitoring and collecting information about several aspects of the activity (measure movement-based parameters such as distance, velocity, foot strike, acceleration), and capturing physiological signals, such as heart rate, temperature, oxygenation, blood pressure or electrodermal conductivity [4].

The use of wearables in the field of sports mainly focuses on tracking, analysis and improvement of performance, reducing injuries or controlling fatigue. These solutions usually obtain information about the health parameters of athletes [6] or detect postural or physical problems in the performance of the activity [7,8]. However, to achieve these issues, most of the commercial devices only capture and provide tracking information and performance measurements that are processed later and downloaded and analysed by the athletes or their coaches. They are not used to give feedback in real-time since data are processed offline [6] and visualised by the user when the activity is over in order to understand how the training evolved, what problems arose during that practice, what she/he felt in some specific moments, and how that could affect their performance. It has been demonstrated that emotions have a significant impact on our daily lives, on our work, studies, choices or our ability to learn or make decisions. Emotions also affect our health [2] and are a very important aspect in sports performance [9]. Athletes can use different strategies to regulate their emotions and therefore improve their performance [10]. For several years now, there have been many works that have studied and proved that emotional factors are fundamental to athletes’ training, performance and competition achievements [11,12]. Despite these studies, the emotional states of the athletes have been measured in a very limited way, usually analysing the behaviour after performing the activity and through self-assessment questionnaires about their mood [13], which they use to fill in before and after the activity [7]. There is very little research about the emotional states felt by the athletes during the practice of the activity, in real-time and in the wild.

One of the most common methods for automatically recognising emotions is based on capturing physiological signals [14] since these data allow us to reflect the real emotional state of a person in an objective manner, [4] and it is impossible to mask or suppress biosignals representing effects [15]. Among the many physiological signals that provide information about human emotions, the most useful for emotion recognition that can be measured by sensors are: temperature, electrodermal activity, heart rate, blood volume pulse, muscle electrical activity, respiration, and brain electrical activity [14]. The use of these signals to detect and predict emotions using artificial intelligence algorithms is now widely used. However, since measures for expressing emotional states have limitations as subjective indicators, different emotional representations have been proposed, highlighting the following two models: categorical models, in which emotions are divided into discrete categories, such as happy, sad, etc., and dimensional models, in which categories are based on arousal and valence dimensional spaces [16].

In the sports field, wearables technologies are the devices most widely used for detecting emotions because of their ability to obtain data continuously, in real-time and in a non-invasive or intrusive way [6]. Despite the proven importance of emotions in sports performance, and the widespread use of wearables in sports, very few studies have analysed athletes’ emotions in real-time. Usually, these studies use wearables to detect physiological signals for future analysis, offline, but they do not give feedback to the athletes about their emotions during the course of the activity. This is the case of the works of Azhar [17] and Havlucu [18], who use different commercial wearable devices to capture physiological signals that allow to predict the psychological states of tennis players based on coach observations and machine learning algorithms. Bi et al. [19] also used commercial wearables but to recognise the emotional state of long-distance runners during a race in order to create affective maps that are shared with the spectators. The works of Dupre are focused on the use of wearables for modelling emotional patterns in athletes during physical activities [20] and for analysing the relationship between their emotions and their performance when performing zipline activities [21]. There are also many works that use wearables and consider athletes’ emotions to detect stress [22], pain [23] or fatigue perception [4,24].

However, in order to improve athletes’ performance, tackle their boredom or reduce their fatigue and extenuation, it is very important to detect emotions and try to change them during the development of the activity. Among the different ways to motivate athletes, music is one of the most used. For many years now, it has been shown that the effects of music are highly correlated with emotions. Neuroscientific studies have empirically demonstrated that music has the potential to alter mood as it is able to activate the emotional structures of the brain, and these emotions provoke physiological alterations that act on the central nervous system [25,26]. Due to this fact, music has been applied in many types of therapies to regulate emotions [27] to reduce anxiety, stress or depression [28], improve mental health and wellbeing [28], memory [29], and motivation [30]. In many works, music is used to induce or alter emotions in users [31,32], while in other works, the emotional perception of users while listening to music is analysed [33,34]. As in the case of physiological sensors, the same emotional models are used for emotion recognition in music: categorical and dimensional models [35].

Different benefits of music listening for athletes have also been demonstrated, such as control of arousal, reduced perceived effort, and improved performance, among others [36,37,38]. Of all sports, the use of music has become very popular in running due to the fact that it covers everything from novices to professionals [39,40]. Running with music can help to increase the runner’s motivation, making hard training sessions much more pleasant as well as making the runner feel less alone. These effects are of special interest for long-distance runners and also for people with a sedentary lifestyle who wish to start running [41]. For several years now, there have been works that use wearables to detect different physiological data in order to study the influence of music on motivation to exercise [42,43,44] or to improve the runners’ performance [37,45]. Even though, in many cases, the authors talk about detecting emotions, they do not deduce the emotions that the user feels from the physiological signals, in part because the commercial wearables are too expensive. Instead, they use them to capture data such as heart rate to relate it with the rhythm of the music in order to generate an acceleration, maintain a certain speed, achieve a specific step cadence or attain a flow [36].

As part of the *DJ-Running* project, we developed a service-based system for improving the motivation and performance of long-distance runners through *Spotify* music [46]. The system selected and played music according to the runner’s emotions, the characteristics of the training session, and the environment in which she/he was training. From a technological point of view, the solution consisted mainly of three software systems. Firstly, a music emotion recognition system was used, called *RIADA* [47], which determined the emotions that the listeners feel when listen to a song. This system processed the catalogue of *Spotify* songs and created a set of labels that describe the emotional effects each song provokes to the listeners. Secondly, an emotion-based music recommender was used, which interpreted the runners’ context to determine the songs to be played during the training session [46,48]. These recommendations considered affective criteria based on the emotions produced by the songs (and described by the *RIADA* labels) and the emotions felt by the runners. Finally, a mobile application was used that interacted with the recommender to play the recommended songs. The innovation of the *DJ-Running* system resides in the possibility of adapting the music to the emotions that the runner is feeling at each instant, her/his emotional changes and the geographic characteristics of the environment in which she/he is running. This disruptive factor requires recognising and interpreting the runner’s emotions in real-time, for example, using wearable technology. In [49], we sketched the earlier ideas regarding the design of a wearable that could provide the required support for this type of emotion recognition.

In this paper, we focus on the final development of a prototype of wearable that is easily worn by long-distance runners, and the artificial intelligence models programmed to recognise emotions from the data acquired by the sensors integrated into the wearable. Our wristband prototype allows monitoring runners’ physiological activity during training sessions in real-time. In particular, we are interested in monitoring the runners’ certain skin responses. These physiological responses have been widely used in the problem of recognising emotions [50,51,52]. Then, different machine learning algorithms were evaluated and compared between them to build a recognition system from the signals obtained by the wearable. The best recognition model is selected to be integrated into the *DJ-Running* mobile application in order to support the process of recommending music based on the runner’s emotions. The main contributions of the recognition system presented in this paper with respect to the existing approaches are:

- The wearable has been developed using low-cost sensors and electronic components that are easy to find on the market. This fact and its simple design make the device easily replicable.
- The runner’s ergonomics have been studied in order to design a wristband that is comfortable and convenient for sports use. In this design, the placement of the sensors ensures that their measures are reliable during the physical activity.
- The wearable’s hardware acquires measurements from the sensors in real-time and allows access to the raw data (through a USB connector or the Bluetooth communication network). Most of the existing devices are sold in conjunction with software applications that provide access to processed information but not to the raw sensor data (for example, *Empatica E4* [53], the most popular wearable in the field of the affective computing); other commercial products allow access to raw data but are not intended to be used by a user on the move, such as GSR Loger [54], Plux [55] or Shimmer [56].
- A procedure for processing the raw wearable data in order to characterise the runner’s physiological response during the physical training is defined and programmed. This characterisation is then used to deduce the runner’s emotions by applying artificial intelligence techniques.
- The wearable and emotion recognition models are integrated to provide a prototype of product intended to develop affective mobile applications. The result allows the recognition of emotions in real-time, even when the user is in motion, as an alternative to the more commonly used methods based on self-assessment questionnaires.
- The solution has been tested in the context of the *DJ-Running* project to adjust the music recommendations to the runner’s real-time emotions.

This paper is organised as follows. Section 2 describes the design of the wearable for runners. Section 3 presents the process of building the machine learning models used for recognising runners’ emotions. It includes the filtering of data obtained through the wearable, the representation of emotions, the extraction and selection of features, and the application of different recognition strategies based on machine learning techniques. The integration of the resulting models into the *DJ-Running* mobile application is then described in Section 4. Finally, some conclusions and future work are detailed in Section 5.

## 2. Description of The Wearable

**Figure 1:**
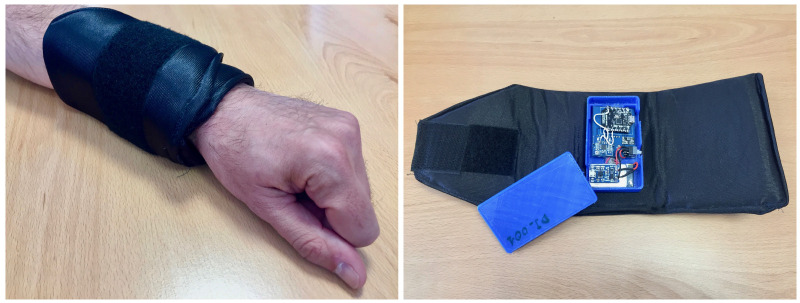
Bracelet prototype and its hardware components.

The design of the wearable must be lightweight and not hinder the athletes when performing physical exercise. For this reason, it has been built as a bracelet, as shown in Figure 1. The bracelet is made of a textile wrist support with Velcro straps for easy placement and an inner box that incorporates the electronic components of the device.

**Figure 2:**
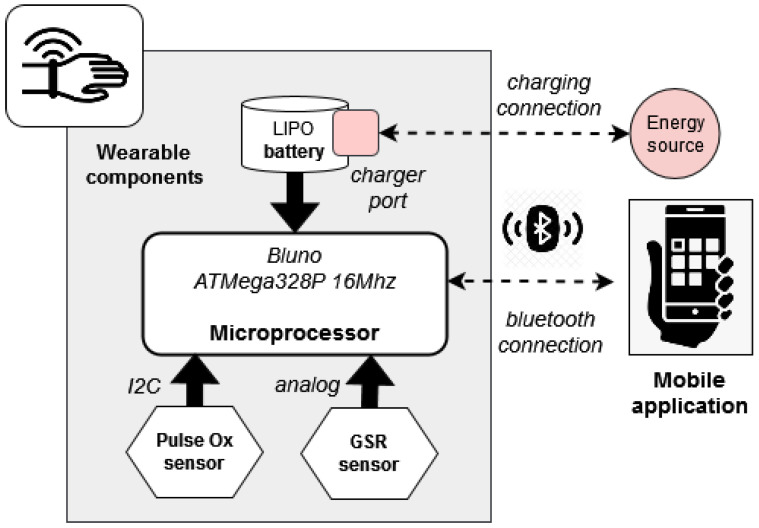
Hardware components integrated into the bracelet.

Figure 2 depicts the main components of the designed hardware. The bracelet integrates a Galvanic Skin Response (GSR) sensor for measuring the electrodermal conductivity of the skin. GSR data reflect emotional arousal and are usually used in the recognition of emotions based on physiological wearables [57]. The GSR sensor is located in the inside part of the wrist. This location is validated as useful for measuring GSR data [58]. Internally, the sensor consists of two nickel contacts and an instrumentation amplifier that provides an analogue signal, which is read by the integrated ADC converter of the wearable’s microprocessor. The microprocessor is also able to send the monitored data to a mobile application through a Bluetooth Low Energy (BLE) protocol.

We have selected the Bluno Beetle BLE board with an ATMega328P@16Mhz microprocessor and Bluetooth capability integrated into the board with the TI CC2540 chip. This microprocessor board is one of the smallest Arduino BLE boards on the market. It has four analogue input pins, four digital pins and one I2C port. The transmission distance of the Bluetooth communication is 30 m (50 m in an open field). This module is compatible with the standard Arduino IDE program to upload codes without any extra libraries or drivers.

A battery-operated system is mandatory for a wearable system such as the developed bracelet. In this case, we have selected a rechargeable LiPo battery with 500 mAh/3.7V capacity. We have included an electronic board based on the TP4056 charger. This board is a complete constant-current/constant-voltage linear charger module with a protection circuit. The module has two LEDs, red and green, to show charging in progress or charge termination, respectively.

The information obtained is stored in RAW format and provides the resistance data obtained from the sensor and a timestamp that allows for identifying the events in the emotion detection experiments.

## 3. Machine Learning Models for the Recognition of Emotions

This section begins by introducing the existing models for representing emotions. One of them has been selected for building the recognition models that deduce runners’ emotions from their physiological data. The process of building involves the creation of an affective dataset, the extraction and selection of features and the performing of machine learning models.

### 3.1. Representation of Emotions

Firstly, it is necessary to introduce the models for representing users’ emotions. As it was previously mentioned in Section 1, two different types of models have been widely used in the field of affective computing: categorical and dimensional models. This work is based on the Russell’s circumplex model [59], one of the most popular dimensional models. It represents affective states over a two-dimensional space that is defined by the *valence* (X-axis) and *arousal* (Y-axis) dimensions. The valence represents the intrinsic pleasure/displeasure (positive/negative) of an event, object or situation, and the arousal represents the perceived intensity of a feeling from very calming to highly exciting or agitating. The combination of these two dimensions (valence/arousal) determines four different quadrants: the *aggressive* (negative/positive), the *happy* (positive/positive), the *sad* (negative/negative) and the *relaxed* (positive/negative) quadrant. Then, each emotion is mapped to a point in the two-dimensional space and, therefore, is also located in one of the mentioned quadrants.

### 3.2. Creation of a Physiological Dataset

Once a model for representing emotions is selected, the following step consists of creating the physiological dataset that will be used to build the recognition models. This dataset must contain information that helps us to understand which is the runners’ physiological response to an emotional stimulus. In the context of the *DJ-Running* project, the music is used as a stimulus to provoke emotional changes that have a positive effect on the runners. Therefore, music, emotions and wearable technology have been combined in order to create the necessary dataset.

An experiment has been made to determine the physiological response of the users when listening to songs that provoke certain emotions of interest. These responses are monitored through the wearable and used for creating the dataset. The method, the instruments and participants involved in the experiment, and the obtained results are described in the following paragraphs.

*Methodology*: The experiment was conducted in a room without noise disturbances in which the participant is lying on a stretcher in a state of rest. It consists of playing a set of songs that provoke different emotions and monitoring the listener’s physiological response through the wearable previously described. The physiological data acquired through the wearable during the playing of the songs are stored in the internal memory of the device. Those data can be downloaded via a USB port once the experiment is finished.

*Equipment*: Four instruments were used during the experiment: the wearable for recording the listener’s physiological response, an affective playlist that contains the songs to be played, an MP4 player for playing those songs, and a pair of headphones for listening to them. The playlist programmed consists of nine songs, two belonging to each of Russell’s emotional quadrants (specifically, to the *Happy*, *Sad*, *Aggressive* and *Relaxed* quadrants) and another extra song used at the beginning of the experiment to relax the listener. Pauses of 15 seconds have been introduced between consecutive songs to avoid the emotional response of two different songs potentially overlapping. The total duration of the playlist is approximately 41 minutes.

*Participants*: The total number of participants in the experiment was 32. All of them were men between 25 and 45 years old. They were randomly chosen from a register of athletes and participated in the experiment on a voluntary basis. The participants have a healthy lifestyle and regularly practise sports (an average of five hours a week). Before starting the experiment, they declared that their mood was usual (none were occasionally stressed or nervous, for example) and that none of them suffered from emotional disorders that could have an influence on the results. *Results*: The wearable generates a physiological data file for each session. It contains the listener’s response to the nine songs, specifically, the data acquired from the EDA sensor. Therefore, the raw result of the experiment was 32 files of physiological data (one for each listener). Nevertheless, a *validation procedure* was applied to discard those files in which the data acquired through the wearable contained errors or were incomplete (specifically, 7 files were discarded). Once that procedure was concluded, each of the 25 valid files was split into several pieces, so each one stores the data recorded during the playing of a specific song. In particular, we were interested in obtaining eight data files, two relating to happy songs, two to sad songs, and so on for the rest of Russell’s emotional quadrants (the song used at the beginning of the experiment for relaxing the listener was discarded). In addition, an emotional label was assigned to each of these files for characterising the emotion induced in the listener through the corresponding song (thus, the *Happy*, *Sad*, *Aggressive* and *Relaxed* labels were used). After completing this process, the experiment’s target dataset was created from the 200 files of EDA data obtained (50 files per emotional quadrant). These files and their labels are the samples that will be then used to create the recognition models based on machine learning techniques.

### 3.3. Sensor Signal Processing and Feature Extraction

**Figure 3:**
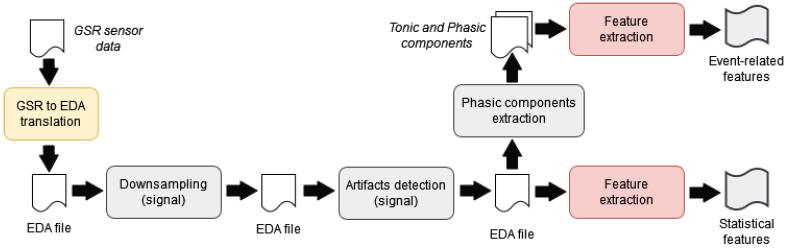
Stages involved in the feature extraction from the files of sensor data.

Figure 3 shows the process of extracting the features that will be used for building the emotion recognition models from the files of physiological data. Similar processes have been applied in other proposals that work with this type of sensor data [60,61,62,63,64]

The process of feature extraction is applied individually to each data file resulting from the experiment previously described. At the beginning of the process, the *GSR to EDA translation* step converts the measurements acquired by the GSR sensor to electrodermal activity values (EDA). Both measures serve to reflect emotional arousal, but GSR measures the skin electrical resistance in ohms and EDA the conductivity in siemens. This conversion has a twofold purpose. On the one hand, it allows the calculation of features based on the analysis of the EDA signal peaks. These features have been widely used in the problem of emotion recognition. On the other hand, given the existence of commercial wearables integrating an EDA sensor (for example, the *Empatica E4* wristband [53]), an emotion recognition system based on those values could be highly reusable and able to work with physiological data from different devices.

Then, the *downsampling* of EDA data is applied to reduce the sample rate of the signal, two samples per second, without loss of information. This reduction facilitates the subsequent processing of the signal. The following step, called *artefact detection*, consists of removing the noise of the output EDA signal. It applies a median filter, a noise reduction technique usually used in signal filtering, to improve the quality of the data.

Some features can be directly extracted from the filtered signal, while others require the analysis of the signal’s context. In [52], these features are named *statistical features* and *event-related features*, respectively. As part of the process, a set of statistical features are calculated from the result of the artefact detection. These features are listed and explained in Table 1. On the other hand, the *Phasic components extraction* step is responsible for analysing the signal’s context in order to extract the event-related features. It consists of calculating the tonic and phasic components of the signal. Intuitively, the tonic component represents the conductivity level of an individual in a context (the slow changes in the signal), whereas the phasic corresponds with the individual’s responses to a specific stimulus (the fast changes). The phasic component is derived from the tonic and is used to detect relevant changes (peaks and offsets) from a physiological point of view. As was proposed in [65], these changes must have a slope of ±0.01 microsiemens per second and a duration of 3 s. These parameters could vary according to the individual or to the activity that she/he is doing but are considered adequate in the general case. Once the changes are calculated, these are processed for calculating the event-related features of interest. These are shown in the bottom part of Table 1.

Finally, these features have been normalised in order to make their scales of values uniform. This normalisation is necessary for the application of certain machine learning algorithms, for example, for algorithms that use distance measures such as k-nearest neighbours. We used the *Scikit MinMaxScaler* tool [66] to transform the features’ scales, with 0 being the minimum value and 1, the maximum value of each one.

### 3.4. Selection of Features

As part of the work, different classification models have been built in order to determine the best option for recognising the runner’s emotions. These models will deduce the emotions (the model’s output) from the physiological data acquired through the device that the runner wears (the model’s input). The following two models have been considered:

- Option 1: A multiclass classification model that assigns the input sample to exactly one of Russell’s emotional quadrants (a 4-class model). The output is a vector composed of four pairs of values (a logical value and a real value), with each pair representing the probability that the emotions felt by the athlete are located in the corresponding quadrant. For example, the output ((*true*, *false*, *false*, *false*), (0.84, 0.18, 0.12, 0.06)) represents a *happy* emotion with a 0.84 probability. The sad, aggressive and relaxed probabilities (0.18, 0.12 and 0.06, respectively) are lower than the classification threshold, and therefore, the input is also classified as not sad, not angry and not relaxed.
- Option 2: Four binary classification models, one per each Russell quadrant, predict whether (or not) the emotion that the runner feels at a particular moment in time belongs to the corresponding quadrant. Therefore, the output of each of these classifiers is a pair of values (a logical value and a real value). For example, in the case of the *Happy* classifier, the result (*false*, 0.2) represents that the user is not feeling an emotion located in the happy quadrant (similar to the rest of the models).

Before building these models, it is necessary to identify the features that will be involved in that process. In option 2, in which more than one model will be created, the features are analysed from the perspective of each particular model; that is, we are supposing that a feature may be significant for recognising the output of a classifier but irrelevant to others.

Certain statistical tests frequently used in machine learning have been used for evaluating and interpreting the relevance of the features. In particular, we have selected three different statistical tests for evaluating the degree of features’ relevance in each of the classification options: the *Chi square*, *ANOVA F-value* and *Mutual information* tests. These tests are usually applied jointly to solve classification problems because they find the features “most related” to the model’s output from different points of view [52]. Regardless of the interpretation of “most related”, each test orders the features from most to least relevant, and then the corresponding results are combined, applying a voting strategy.

Table 2 shows the result for each classification option after applying that multi-test approach based on voting. The first column presents the features, ordered from most to least relevant, involved in the building of the multiclass model (*4-Class*) described in option 1. The rest of the columns with the *Happy*/*Sad*/*Aggressive*/*Relaxed* models of option 2. The results provide evidence that the usefulness of the features varies according to the classifier to be built. As part of this building process, a feature selection method will be applied to reduce the set of available attributes and create more accurate classifiers.

### 3.5. Building of the Recognition Models

The goal is now to build the described classification models and compare their performance in order to select the best approach for the recognition of emotions. Five types of machine learning algorithms have been considered: *K-nearest neighbours* (KNN), *random forest* (RF), *multi-layer perceptron* (MLP), *linear support vector classifier* (LSVC) and *gradient boosting* (GB). Some of these algorithms have been previously used with good results in the recognition of emotions [60,62,67]. Nevertheless, we have also considered the possibility that the use of a unique algorithm is not the best option for building the different classification models. Therefore, the best machine learning algorithm for each particular model is also studied.

Before comparing the different algorithms, the candidate models have been trained by applying different permutations of features and different configurations of hyperparameters in order to find the best option from a classification point of view. The evaluation of configurations has been carried out by using the *randomised search* algorithm provided by *Scikit Learn* [68] and the training by performing a *Repeated 5-fold cross-validation*. The use of this validation approach is especially important when the models are built from small-sized datasets, as in this case, because it avoids making the test with poorly representative samples. The repeated cross-validation reduces the overfitting error derived from working with these small-sized datasets and improves the performance of the models.

Table 3 shows the results obtained for the different classification options (4-Class and Happy/Sad/Aggressive/Relaxed, respectively). For each of them, the five machine learning algorithms have been applied in order to build the corresponding recognition model (second column of the table). These models have been configured with the optimal input of features (third column of the table) and hyperparameters. Then, four of the classification metrics usually used in the evaluation of models have been calculated for the different solutions: *Accuracy*, *F1*, *Precision* and *Recall* (from the fourth to the seventh column). We are especially interested in analysing the F1-score results, which are calculated from the precision and recall metrics. The F1-score provides a reliable measure when the dataset used for the creation of models is unbalanced, as in this case. The dataset used in this work contains 25% of positive samples (the emotion to be recognised) and 75% of negative samples (the rest of the emotions), and therefore, the accuracy score could provide an over-optimistic estimation of the classifier ability on the majority class. Finally, after analysing the classification metrics, the best machine learning model has been highlighted in green colour.

In the case of the multiclass classification model (4-Class, Option 1), the F1-score results of the five models that were built are very poor. The *random forest* model stands out slightly from the others, with an F1-score of 0.288, but in general, the results are very similar (these seem to be correlated with the number of classes to be recognised by the models). Therefore, the recognition capacity of these models is lower than desirable for determining the user’s emotions.

In the case of option 2, the use of a different machine learning algorithm for recognising the emotions belonging to each Russell quadrant seems to be a good option; more specifically, the *k-nearest neighbours* models have been selected for the recognition of the *Sad* and *Relaxed* quadrants and the *random forest* models for the *Happy* and *Aggressive* quadrants. The F1-score of the selected models has a value between 0.61 and 0.65. The results of this second option improve those obtained in the 4-Class approach. Although these results can seem low with respect to what is desirable in the machine learning problems, they are similar to the results obtained in other emotion recognition proposals based on physiological data [60,62].

## 4. DJ-Running: An Emotion-Based Application for Recommending Music

The goal of the *DJ-Running* project is to increase runners’ motivation through *Spotify* music. The wearable and the machine learning models presented in the previous sections are used to determine runners’ emotions during their training sessions. These emotions are then analysed to select the songs to be played at each moment.

**Figure 4:**
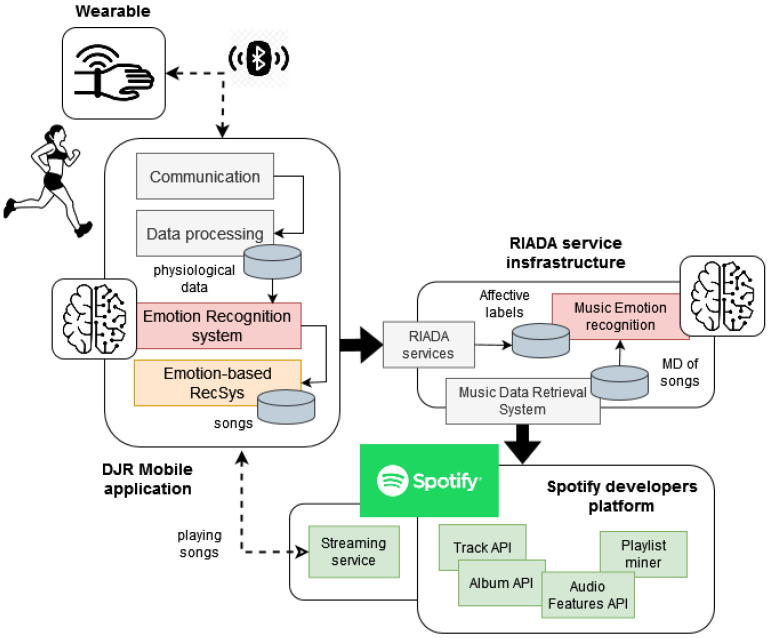
Technological components involved in the *DJ-Running* system.

Figure 4 shows a high-level overview of the technological components involved in the final solution. As is shown on the left side of the figure, the runner’s equipment consists of the wearable and a mobile application that plays the recommended songs during the training session. The wearable monitors the runner’s physiological parameters and sends them periodically to the application through a *Bluetooth* connection. The application internally processes these sensor data to deduce the runner’s emotions, determines the songs to be played according to those emotions and connects with the *Spotify* streaming service to play them.

The application is composed of four software modules: the *communication* component, the *data processing* component, the *emotion-based decision-maker* system and the *emotion-based music recommender*. The two first components are responsible for managing the wireless communication with the wearable, receiving the sensor data and filtering those data before storing them in a local repository. The filtering is based on the process described in Figure 3 and aims to extract the corresponding features (statistical and event-related features) from the received data. The other two modules are responsible for recognising the runner’s emotions and for playing motivating music based on those emotions, respectively. The technical details of these two systems are described in the following paragraphs.

The *emotion-based decision-maker* system integrates the four binary classification models described in Section 3. These models determine the emotion that the runner is probably feeling at a specific time interval (each 30 seconds) from the wearable data. For example, the output ((*true*, *false*, *false*, *false*), (0.71, 0.03, 0.13, 0.33))) represents that the runner feels *happy* with a probability of 0.71. The sad, aggressive and relaxed probabilities (0.03, 0.13 and 0.33, respectively) are lower than the classification threshold, and therefore, the runner’s feeling is classified as not sad, not aggressive and not relaxed. The sequence of emotions that is recognised during the training session allows the system to define the runner’s mood and the emotional changes that she/he suffers during that time. That affective information is then used to determine the emotion to be induced in the runner through the music. For example, when the runner is stressed, the system should decide that the songs to be played should be relaxing. Then, once she/he is relaxed, the system will suggest that the songs should be motivating in order to produce a positive effect in the runner’s mood. These emotion-based decisions are made by a rule-based engine. It integrates a set of rules that were defined in collaboration with runners as part of the *DJ-Running* project. A complete description of the method used to determine these rules can be found in [46].

The *emotion-based music recommender* determines the specific songs to be played by the mobile application from the emotion suggested by the decision maker. The recommender requires a database of songs that have been previously labelled from an emotional perspective. In this work, this database has been created (and periodically updated) from the services available in the *RIADA infrastructure* [47]. RIADA offers a repository of affective labels that determine the emotion that each *Spotify* song will probably produce in the listener (more than 50 million songs have been labelled).

The right side of Figure 4 provides an overview of the RIADA components that were involved in the creation of labels. These labels are also based on Russell’s affective model and were determined from songs’ metadata and audio features. A *music data retrieval system* interacts with the service-oriented platform of *Spotify* in order to access its catalogue of songs and the metadata and audio features of these songs. Then, a *music emotion recognition* system automatically determines the emotion produced by each song from its audio features. These emotions are translated to labels that are stored in the database of the RIADA infrastructure. The music emotion recognition is based on machine learning techniques and has been programmed to support large-scale annotation processes, as was detailed in [47]. Finally, the infrastructure publishes an API that allows applications to access its database of labelled songs.

The core of the *emotion-based music recommender* is a *nearest neighbours algorithm* that works on the local database of labels to find the candidate songs to be played. The recommender creates a *search space* from the songs’ audio features and emotional labels contained into the database. Each point of the space corresponds with a concrete song. When a new recommendation request is received, the recommender translates the input emotion (the emotion to be produced by the runner) to a *search point* in the space. This translation is calculated by applying regression models based on the songs’ audio features and the effects that some of these features can produce in the listeners from an emotional point of view. Then, the neighbourhood algorithm is applied to find a set of candidate songs (songs close to the search point). These candidates are subsequently filtered to discard similar songs or to remove songs that do not fit the runner’s preferences. These preferences are defined by the application’s user during the installation process and include basically the music genres, styles and decades of interest.

## 5. Conclusions and Future Work

In this paper, a prototype of a product to recognise mobile users’ emotions in real-time has been presented. It consists of a complete result of engineering that combines people, wearables, machine learning techniques, mobile computing and service-based solutions. The solution has been designed to be easily integrated into mobile applications where the affective component of the users plays a relevant role. The emotion recognition based on self-assessment questionnaires has some disadvantages: users are not always able to express how they feel, it can be tedious for users, or it is not applicable for certain types of problems (for example, when users are in continuous motion). For these reasons, the real-time recognition emerges as a very interesting alternative. In addition, the possibility of having access to the raw sensor data allows programming other knowledge models based on physiological data or data mining solutions that help to understand users’ affective behaviour.

From a technical perspective, the solution consists of a wearable and a set of recognition models. The wearable is a prototype specially designed for runners, with simple hardware and low production cost compared to commercial devices. The emotion recognition models are based on machine learning algorithms. Different features have been extracted from the EDA sensor data and evaluated in order to determine their relevance in the process of emotion recognition. Then, two different classification approaches were evaluated: a multiclass classification model versus four binary classification models. The results of the first approach have not been as good as expected after considering different permutations of features and different configurations of hyperparameters. The second approach turned out to be a better option when the *random forest* and *K-nearest neighbours* algorithms were combined for deducing emotions.

The proposed solution has been integrated into the *DJ-Running* mobile application in order to test its applicability to a real problem. In this case, emotions were used to regulate the runners’ mood through the music depending on the physical activity they are performing and the environment in which they are training.

As future work, on the one hand, we are particularly interested in using the results of this research to achieve a solution closer to a product in the medium term. It requires, first of all, refining the wearable hardware to reduce its size and integrating new sensors that are useful to improve the recognition of emotions. In addition, the data of these sensors will have to be processed to obtain new features that allow building more accurate recognition models. On the other hand, we would like to reuse the results for other application domains, for example, health, wellness or entertainment. In these domains, emotions and data from other smart devices (for example, installed in the home or worn by the user) will be likely combined to extract knowledge to help create new user-centric experiences.

## Figures and Tables

**Table 1:** Description of the features used in the recognition.

Statistical Features	
**Acronym**	**Definition**
Mean	EDA mean for each sample
Median	EDA median for each sample
Std	EDA standard deviation for each sample
Max	EDA maximum for each sample
Min	EDA minimum for each sample
Kurtosis	Determines whether the tails of the given EDA signal contain extreme values
Skewness	Determines the asymmetry of the EDA signal from the point of view of a distribution
AUC	Area under the curve of the EDA signal per second
PSD	Power of Spectral Density for EDA signal median
**Event-related features**	
**Acronym**	**Definition**
MeanPA	Mean in seconds of all peaks’ amplitudes
MaxPA	Max in seconds of all peaks’ amplitudes
MeanOA	Mean in seconds of all offsets’ amplitude
MaxOA	Max in seconds of all offsets’ amplitude
PPS	Number of peaks in a time window divided by the duration of the window in seconds
OPS	Number of offsets in a time window divided by the duration of the window in seconds

**Table 2:** Features (explained in Table 1) in order of highest to lowest relevance.

Option 1	Option 2			
**4-Class**	**Happy**	**Sad**	**Aggressive**	**Relaxed**
MaxOA	MeanPA	PSD	PPS	MeanPA
Kurtosis	MaxPA	MaxOA	Kurtosis	MaxPA
MaxPA	Max	Kurtosis	MaxOA	PPS
PPS	PPS	Skewness	MeanOA	MaxOA
MeanPA	MaxOA	MeanPA	OPS	Max
Max	OPS	MaxPA	PSD	Std
PSD	Skewness	AUC	Std	Skewness
MeanOA	Kurtosis	Median	Mean	Median
Skewness	MeanOA	Max	MaxPA	PSD
Std	Std	MeanOA	Median	Mean
OPS	PSD	PPS	Min	MeanOA
Median	AUC	Min	MeanPA	OPS
Mean	Mean	Mean	Max	AUC
AUC	Min	OPS	AUC	Kurtosis
Min	Median	Std	Skewness	Min

**Table 3:** Machine learning models for each classification approach (the best model is highlighted in green color).

Option 1	Model	Nº Features	Accuracy	F1	Precision	Recall
4-Class	KNN	10	0.288	0.277	0.283	0.288
	RF	5	0.291	0.288	0.293	0.290
	MLP	12	0.295	0.284	0.296	0.295
	LSVC	12	0.303	0.279	0.282	0.303
	GB	5	0.273	0.262	0.273	0.273
**Option 2**	**Model**	**Nº Features**	**Accuracy**	**F1**	**Precision**	**Recall**
Happy	KNN	5	0.683	0.569	0.584	0.556
	RF	5	0.728	0.641	0.673	0.612
	MLP	8	0.604	0.525	0.523	0.529
	LSVC	8	0.634	0.602	0.607	0.598
	GB	5	0.681	0.584	0.578	0.592
Sad	KNN	10	0.698	0.612	0.609	0.616
	RF	12	0.712	0.560	0.573	0.548
	MLP	15	0.621	0.494	0.498	0.491
	LSVC	8	0.604	0.555	0.533	0.579
	GB	12	0.643	0.514	0.516	0.513
Aggressive	KNN	15	0.704	0.558	0.578	0.541
	RF	5	0.728	0.654	0.684	0.628
	MLP	15	0.659	0.563	0.567	0.561
	LSVC	10	0.485	0.510	0.509	0.512
	GB	5	0.698	0.588	0.608	0.571
Relaxed	KNN	5	0.709	0.639	0.638	0.641
	RF	5	0.719	0.617	0.634	0.601
	MLP	12	0.640	0.531	0.534	0.529
	LSVC	5	0.554	0.565	0.573	0.558
	GB	8	0.704	0.615	0.628	0.603

## Data Availability

Not applicable.
